# Atypical Dermatophytosis in 12 North American Porcupines (*Erethizon dorsatum*) from the Northeastern United States 2010–2017

**DOI:** 10.3390/pathogens8040171

**Published:** 2019-09-30

**Authors:** David B. Needle, Robert Gibson, Nicholas A. Hollingshead, Inga F. Sidor, Nicholas J. Marra, Derek Rothenheber, Anil J. Thachil, Bryce J. Stanhope, Brian A. Stevens, Julie C. Ellis, Shelley Spanswick, Maureen Murray, Laura B. Goodman

**Affiliations:** 1 New Hampshire Veterinary Diagnostic Laboratory, College of Life Sciences and Agriculture, University of New Hampshire, Durham, NH 03824, USA; David.Needle@unh.edu (D.B.N.); Robert.Gibson@unh.edu (R.G.); Inga.Sidor@unh.edu (I.F.S.); bstev@uoguelph.ca (B.A.S.); 2 Animal Health Diagnostic Center, Cornell University, Ithaca, NY 14853, USA; nah88@cornell.edu (N.A.H.); nmarra@drury.edu (N.J.M.); derek.rothenheber@gmail.com (D.R.); anil.thachil@ncagr.gov (A.J.T.); bjs288@cornell.edu (B.J.S.); 3 Northeast Wildlife Disease Cooperative, Cummings School of Veterinary Medicine at Tufts University, Grafton, MA 01536, USA; jellis04@vet.upenn.edu; 4 Center for Wildlife, Cape Neddick, ME 03902, USA; shelley@thecenterforwildlife.org; 5 Wildlife Clinic, Cummings School of Veterinary Medicine at Tufts University, Grafton, MA 01536, USA; Maureen.Murray@tufts.edu

**Keywords:** dermatophyte, porcupine, *Erethizon*, fungus, metagenomics, fungal genetics, molecular diagnostics

## Abstract

Twelve wild North American porcupines (*Erethizon dorsatum*) out of a total of 44 of this species examined in an 8-year period were diagnosed with dermatopathies while being cared for at two wildlife rehabilitation clinics. Biopsy and necropsy were performed on seven and five animals, respectively. Atypical dermatophytosis was diagnosed in all cases. Lesions consisted of diffuse severe epidermal hyperkeratosis and mild hyperplasia with mild lymphoplasmacytic dermatitis and no folliculitis. Dermatophytes were noted histologically as hyphae and spores in hair shafts, and follicular and epidermal keratin. *Trichophyton* sp. was grown in 5/6 animals where culture was performed, with a molecular diagnosis of *Arthroderma benhamiae/Trichophyton mentagrophytes* in these five cases. Metagenomic analysis of formalin-fixed paraffin-embedded tissue samples from three cases identified fungi from 17 orders in phyla Basidiomycota and Ascomycota. Alteration of therapy from ketaconazole, which was unsuccessful in four out of five early cases, to terbinafine or nitraconazole led to the resolution of disease and recovery to release in four subsequent animals. In all, six animals were euthanized or died due to dermatopathy, no cases resolved spontaneously, and six cases were resolved with therapy. The work we present demonstrates an atypical lesion and anatomical distribution due to dermatophytosis in a series of free-ranging wild porcupines and the successful development of novel techniques for extracting and sequencing nucleic acids from fungus in archival formalin-fixed paraffin-embedded animal tissue.

## 1. Introduction

The North American porcupine (*Erethizon dorsatum*) is the second largest rodent in North America, inhabiting forested lands of the continent whose name it bears from Mexico to Canada, reaching past the tree line in Canada and far into Alaska [1]. The North American porcupine (NAP) has a lasting effect on the timber of their home range as routine annual feeding on the bark of the crown of a tree during a potentially decades-long life results in short, stocky trees with irregular low crowns and clumped young shoots emanating at odd angles. The collection of such “witch trees” and the poaching of fruit from orchards was in part the impetus for the systematic hunting of porcupines including bounties that continued well into the second half of the 20th century in some states [1]. Though slow-moving, near-sighted, and not particularly nimble, the prodigious physical barrier of porcupines wards off most predators, save mountain lions (*Puma concolor*) and fishers (*Martes pennanti*) [1]. The survival and prevalence of this animal is all the more impressive when their poor vision and slow pace are combined with their penchant for falling from trees while foraging and sustaining significant musculoskeletal injuries [1].

Aside from trauma, previously described diseases of porcupines include papillomaviral cutaneous papillomas, fatal toxoplasmosis, portal and hepatic schistosomiasis due to *Schistosomatium douthitti*, *Frenkelia* sp. encephalitis, hepatic *Capillaria hepatica* infection, orchitis with intralesional *Histoplasma*-like organisms, hepatic lipidosis, encephalitis from *Baylisascaris procyonis*, and quill dissemination and sepsis due to fetal death with a concurrent choriocarcinoma [2,3,4,5,6,7,8,9,10,11,12]. There is also a report of a family of NAP housed in a zoological collection in Japan with *Arthroderma benhamiae* infection as well as a recent description of diagnosis and successful medical treatment of *Microsporum gypseum* infection in a single zoo-housed NAP in Kansas [13,14]. While these two reports demonstrate the possibility of NAP in zoos having dermatophyte infections, to our knowledge, there has been no report of such infections in wild NAPs. The purpose of this study was to describe a novel presentation of dermatophytosis in twelve wild NAPs from Massachusetts and Maine occurring from 2010–2017. In addition to fungal culture, the initial development of clinical fungal metagenomic sequencing of the fixed samples is presented to facilitate further characterization of archived cases for future epidemiologic and clinical investigations. 

## 2. Materials and Methods

Cases: No experimental animals were used in this study. The twelve cases included in this report were among 44 NAPs submitted to the New Hampshire Veterinary Diagnostic Laboratory (NHVDL) from 2010–2017 for routine clinical diagnosis. Eleven animals were presented to the Center for Wildlife (Cape Neddick, ME, USA) and one animal was presented to the wildlife clinic at the Cummings School for Veterinary Medicine at Tufts University (North Grafton, MA, USA). Clinical signs at admission included dermatopathy that was considered consistent with mange. Formalin-fixed tissue sections were sent to the biopsy service at the NHVDL in all cases, with plucked hairs submitted to the microbiology section at the NHVDL for culture in six cases. 

Histopathology: Tissues were received fixed in 10% neutral buffered formalin, and then trimmed, processed, embedded in paraffin, sectioned at 5 µm thicknesses, mounted on charged slides, and stained routinely with hematoxylin and eosin. Serial sections were stained routinely with periodic acid-Schiff, or Gomori methenamine silver staining to highlight fungal organisms.

Gross pathology: Five animals (1, 2, 3, 6, and 8) were submitted for full necropsy. Animals were routinely dissected and representative tissue sections were fixed in 10% neutral buffered formalin and processed as described for histopathology. Additional tissue samples were frozen fresh at −20 °C.

Culture: Tissue was submitted to the microbiology section at the New Hampshire Veterinary Diagnostic Laboratory for culture in six cases (3, 8, 9, 10, 11, and 12). Samples included quills, hair, and small skin biopsies. Samples were set up for standard fungal cultures using Sabouraud Dextrose Agar (Northeast Laboratory Services, Winslow, ME, USA) and Dermatophyte Test Media (DTM, Zoetis Diagnostics, Parsippany, NJ, USA). Macroscopic and microscopic assessments were made for a minimum of two weeks. Microscopic preparations were stained using Lacto Phenol Cotton Blue.

Molecular testing: Culture slants from cases 3 and 8–12 and scrolls of formalin-fixed paraffin-embedded tissue (FFPE) from cases 5–7 were submitted to the Cornell Animal Health Diagnostic Center for genetic analysis. Samples were extracted using the DNeasy Plant Mini kit for cultures (Qiagen, Germantown, MD, USA) or the Recover All kit for FFPE (Thermo Fisher Scientific, Waltham, MA, USA). The D1–D2 region of the large subunit RNA gene was amplified by PCR, purified, and sequenced for speciation. Primers NL1 (5′-GCATATCAATAAGCGGAGGAAAAG-3′) and NL4 (5′-GGTCCGTGTTTCAAGACGG-3′) were used for amplification [15]. An additional primer (Seq-NL1-ATCAATAAGCGGAGGAAAAG) was used for Sanger sequencing in cases 6 and 8–12, which was performed at the Cornell University Genomics Facility. Metagenomic sequencing using the same amplicons was performed on cases 5–7, 10, and 12. ITS metagenomic sequencing was additionally performed using primers CTTGGTCATTTAGAGGAAGTAA and GCTGCGTTC TTCATCGATGC on FFPE samples from cases 1 and 7 and cultures from cases 9 and 11. Library preparations for metagenomic sequencing were performed using the Nextera XT library preparation kit and sequenced using MiSeq 2 × 250 bp chemistry (Illumina, San Diego, CA, USA).

Bioinformatics analyses: Reads from the Sanger sequencing were quality trimmed and blasted against the nt database using BLASTn [16] to obtain the top three blast hits at an e-value of ≤ 1e-6. Reads from MiSeq sequencing were demultiplexed into individual libraries for each case. Subsequently, adapter, barcode, and amplification primers (NL1 and NL4) were trimmed from each library along with low quality sequences using cutadapt (v. 1.15) under default settings for filtering and trimming with the exception that the –O overlap setting was adjusted to (-O 5) so that any read with a match to the adapter sequences that had a match of 5 or more bp was trimmed [17]. After adapter trimming, each library was separately assembled with the program SPAdes (v. 3.11.1) using the options for assembling paired end libraries [18]. As a quality control step, we then mapped the input reads from the SPAdes assembly to the SPAdes contigs using the program RSEM (v. 1.2.29) [19], which utilized Bowtie2 (v. 2.3.4) [20] as a short read mapper. We retained all contigs that had 15 or more reads from this analysis and that were between 400 and 700 bp (the expected amplicon size was approximately 600 bp) to remove possible bioinformatic artifacts and contaminating sequences. All contigs that met these criteria were then blasted as described above for Sanger sequences [16]. Any contigs that had hits to a fungal species were retained and the number of reads from the RSEM output for retained contigs were recorded and summed with the number of reads from all contigs that hit identical fungal species to get a proxy for the prevalence of each fungal species in the sample (i.e., a higher read abundance indicates more copies of DNA of the species in the sample, which indicates higher prevalence). The data from the metagenomic sequencing are available at the National Center for Biotechnology Information (NCBI, https://www.ncbi.nlm.nih.gov/) BioProject: PRJNA556991.

## 3. Results

Table 1 summarizes the origin of the animals, signalment, clinical treatment, and case outcome for each animal. Table 2 summarizes the culture, molecular diagnostic, and other pathological findings (when applicable) for each of the 12 porcupines in this study. 

**Figure 1:**
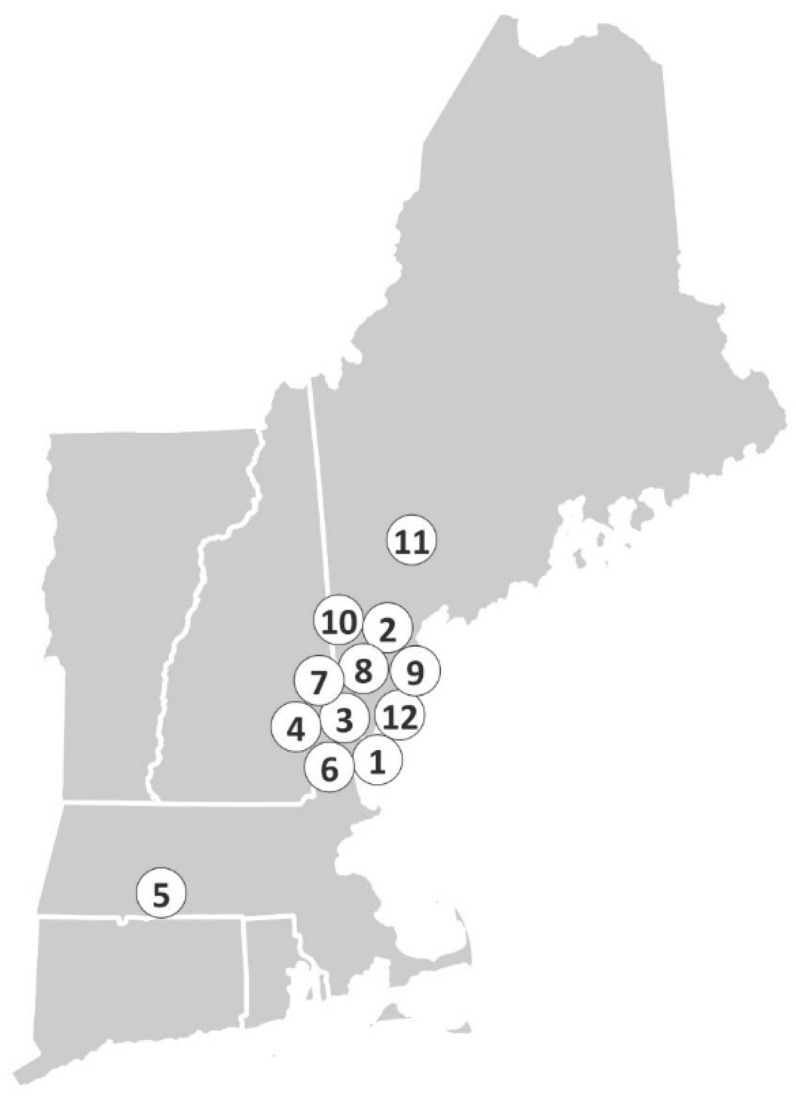
Geographic distribution of cases of dermatophytosis in the 12 wild NAPs in New England. Numbers correspond to case numbers assigned in Table 1.

Cases: All twelve animals were wild, with seven collected in Maine, four from New Hampshire, and one from Massachusetts (Figure 1; Table 1). As could be best surmised by the rehabilitation facility staff, six animals were juveniles and six were adults; nine were male, two were female, and one had no sex indicated. All cases had clinically evident, gross lesions of hyperkeratosis that was suspected to be mange at initial presentation to the rehabilitation clinic. The clinically described lesions were highlighted by hyperkeratosis and had a wide anatomic distribution. Initial treatment varied depending on clinical diagnosis (prior to culture and biopsy) and is summarized in Table 1. 

**Figure 2:**
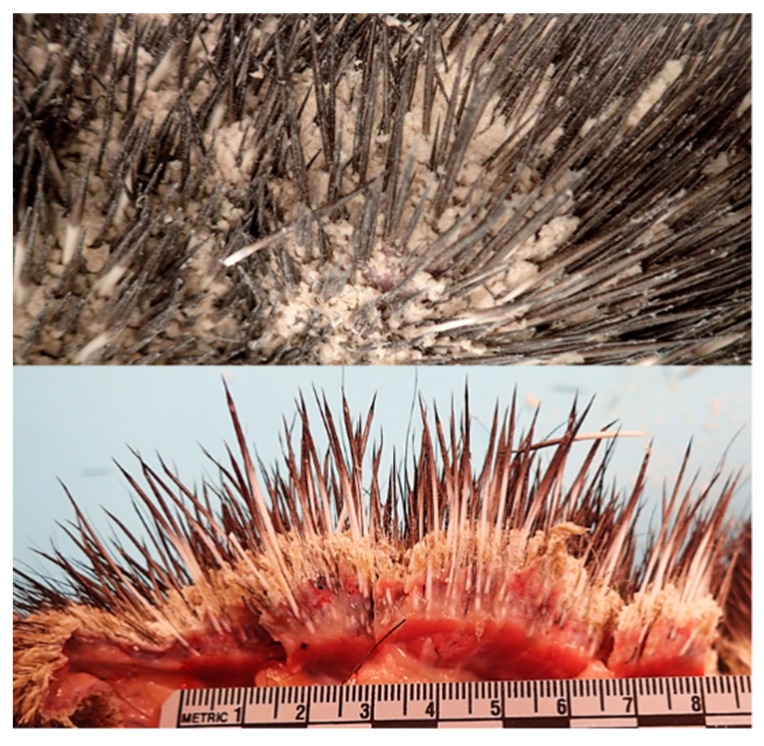
Dermatophytosis in a NAP, animal 8. Gross lesions characterized by severe hyperkeratosis that was readily visible covering the skin on its natural surface and appeared as thick white flaking crusts (**top**); in the cut section the hyperkeratosis was arranged in a thick, diffuse mat of overlapping sheets, measuring up to 5 mm thick (**bottom**).

Gross pathology: The most striking finding in all animals was marked, multifocal (animals 5, 7, 9–12) to diffuse (animals 1–4, 6, and 8), hyperkeratosis and crusting of quilled skin on the dorsum, extending from the face to the tail and onto the proximal aspect of the limbs (Figure 2). Hyperkeratotic crusts were up to 5 mm thick in many regions (Figure 2). There was also patchy hair loss on the ventrum, with mild, patchy, yellow crusting of the skin surface. 

Aside from the lesions of dermatophytosis, marked intestinal cestodiasis was noted in 5/5 animals that underwent necropsy; colonic nematodiasis was noted in 4/5; chronic dental malocclusion in 2/5; and chronic rib fractures in 1/5 animals (Table 2). 

**Figure 3:**
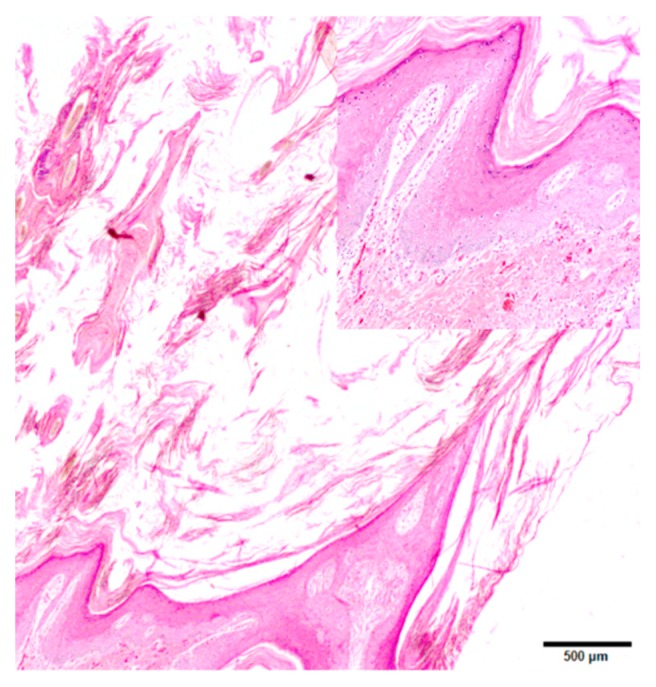
Dermatophytosis in a NAP, animal 8; hematoxylin and eosin. Marked hyperkeratosis extended from the epidermal surface outward, with moderate underlying epidermal hyperplasia. The inset shows minimal/negligible inflammation in the dermis.

Histopathology: Initial diagnosis was made on biopsies submitted to the New Hampshire Veterinary Diagnostic Laboratory for 11/12 animals in the series, with the twelfth animal submitted for necropsy without prior biopsy. The common features of the lesions include a minimal lymphoplasmacytic infiltrate around the superficial dermal vasculature, moderate to occasionally severe epidermal and follicular epithelial hyperplasia, and moderate to severe epidermal and follicular orthokeratotic hyperkeratosis (Figure 3). The epidermal hyperplasia consisted of acanthosis and variable hypergranulosis. There were few to myriad round to oval, 2–6 µm diameter fungal arthrospores colonizing superficial and intrafollicular keratin, the cortices of hair shafts, and in some animals, quills (Figure 3). There were also small to moderate numbers of slender (4–7 µm diameter), undulating, frequently septate, and rarely branching hyphae and arthrospores surrounding some of the affected follicles and invading the cortices of some hair shafts. The fungal organisms were highlighted by periodic acid-Schiff (Figure 3) and Gomori methenamine silver staining. There were few occasions of pigmentary incontinence.

Additional findings in histopathology included chronic cerebrocortical necrosis in 3/5 animals undergoing necropsy; pneumonia in 2/5 porcupines; and subacute to chronic lymphohistiocytic myocarditis in 2/5. Single instances of fibrinonecrotizing phlebitis of liver and esophagus, necrotizing granulomatous peritonitis and cellulitis with intestinal incarceration, hepatitis (presumptive larval), and chronic granulomatous eosinophilic capsular splenitis were also noted.

**Figure 4:**
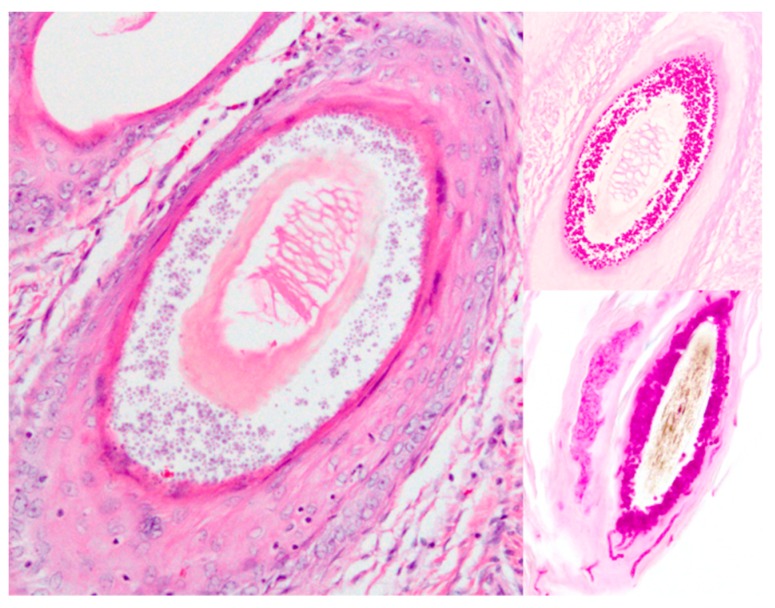
Dermatophytosis in a NAP, animal 8, HE left (larger) image and periodic acid-Schiff highlighting the fungal organisms in both of the inset images on the right. The dermatophyte herein was characterized by round to oval, 2–6 µm diameter fungal arthrospores colonizing the cortices of hair shafts notable in HE staining (large figure, **left side**). The two smaller figures show bright pink periodic acid-Schiff (PAS) staining of the arthrospores (**top right**) as well as slender, 4–7 µm diameter, undulating, frequently septate, and rarely branching hyphae in the follicular keratin (**bottom right**).

**Figure 5:**
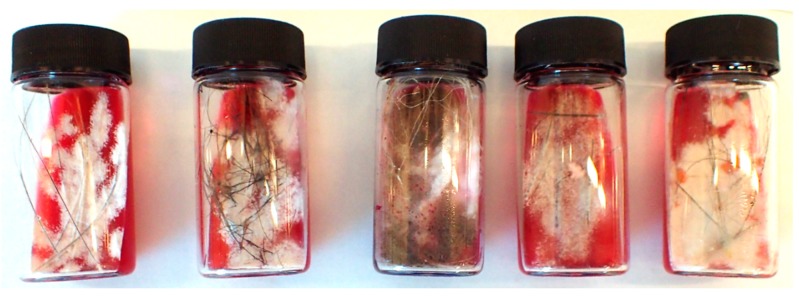
Dermatophytosis in a NAP, five cultures in dermatophyte test medium. From left to right are cases 8–12, respectively. The medium contained a phenol red pH indicator for species producing alkaline metabolites (*Epidermophyton*, *Microsporum*, and *Trichophyton* spp.). Cultures from cases 8, 9, 11, and 12 were characterized by white fungal colonies with powdery surfaces. Case 10 (central) was mixed and characterized by heavy grey-green mold.

Culture: In six (3, 6, 8, 9, 11, 12) of the seven (3, 6, 8–12) specimens submitted for culture, there was growth with a positive color change on dermatophyte test medium (DTM) where these cultures grew white molds that were macroscopically consistent with a dermatophyte (Figure 4). In five of these cases (3, 8, 9, 11, and 12), pure cultures were grown with microscopic morphology that included microconidia and macroconidia consistent with the *Trichophyton* species (Figure 5 and Table 1). The microscopic diagnostic data from case 6 were not recoverable from the case records. The culture in case 10 was mixed and pure subcultures could not be attained.

Molecular analyses: Sanger sequencing of pure cultures obtained from cases 3, 8, 9, 11, and 12 all had *Trichophyton* spp. and/or *Arthroderma benhamiae* as the top three significant hits. The mixed culture from case 10 had a Sanger sequencing result of *Microascus manginii/Scopulariopsis candida*. Sanger sequencing was attempted on the FFPE from case 6, which was of poor quality but yielded significant results for *Alternaria* sp.

**Figure 6:**
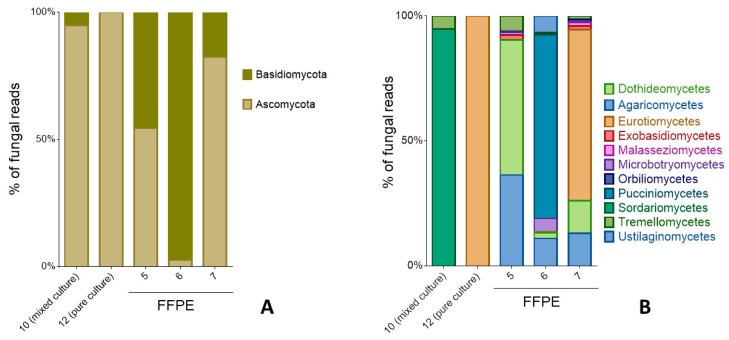
**Fungal composition based on phyla (A) and class (B).** Taxonomic assignments from 28S metagenomic sequencing of mixed or pure cultures were compared with direct sequencing from formalin fixed paraffin-embedded tissues (FFPE).

Metagenomic sequencing was attempted for the cultures from cases 9–12 and the FFPE from cases 1, 5, 6, and 7. Overall, the FFPE samples analyzed by 28S had varying proportions of sequences from the phyla Ascomycota and Basidiomycota (Figure 6A). The class distribution within those orders is shown in Figure 6B. All 28S fungal reads from the pure culture from case 12 belonged to *Trichophyton*/*Arthroderma benhamiae*. Ninety-five percent of the fungal reads from the mixed culture (case 10) were identified as *Scopulariopsis candida*. The other 5% belonged to *Vishniacozyma victoriae*. The FFPE biopsy of case 7 had equal proportions (37% each) of the fungal sequences assigned as *Trichophyton benhamiae* and *Trichophyton mentagrophytes*. 

Based on ITS metagenomic sequencing, the cultures from cases 9 and 11 both had 51% of fungal sequences identified as *Arthroderma benhamiae*. The FFPE biopsies from cases 1 and 7 had 56% and 46% of fungal sequences also identified as *A. benhamiae*, respectively. Case 7 additionally had 3% of fungal reads assigned to *Trichophyton eriotrephon*.

## 4. Discussion

Of the 44 wild or captive porcupine biopsy or necropsy submissions to the NHVDL from 2010–2017, 28 had significant dermatopathy; 12 of these had dermatophyte infection and made up the animals in our report. The primary clinical differential diagnosis was mange, which is commonly reported due to *Sarcoptes scabeie* in NAP, and may also be due to *Notoedres douglasi* [1,21]. With both etiologies, the infection is progressive and often fatal. The degree of hyperkeratosis in the cases we reported was comparable to what was noted in the five cases of mange diagnosed in all 44 porcupines submitted to the NHVDL, however the paws and face tended to be more dramatically affected in cases of mange as compared to the main body mass in dermatophytosis. There were no animals wherein both mange and dermatophytosis were identified. 

In recent years, cutaneous fungi have become some of the most impactful pathogens of wildlife, with white nose syndrome caused by *Pseudogymnoascus destructans* and chytrid fungus infection (*Batrachochytrium dendrobatidis*) decimating bat and frog populations [22,23]. There is evidence that rampant population-level infection with fungi that infect the skin and associated structures are also having significant impacts on human health, with an epidemic of dermatophytosis attributed to members of the same genus (*Trichophyton*) as in our porcupines having spread throughout the subcontinent of India [24]. Dermatophytes are well described in people and animals, although increased globalization has made certain species of these keratinaophilic fungi emergent or re-emergent in certain locals [25]. Dermatophyte hyphae in domesticated animals are morphologically similar to those noted in our NAP, with fungal hyphae in hair and keratin progressing from septation to form arthrospores [26,27]. The predominant fungal isolate in the cultured cases (8–12) was the dermatophyte *Arthroderma benhamiae,* which is the teleomorph of *Trichophyton mentagrophytes*, an obligate parasitic (zoophilic) dermatophyte species [26,28,29]. *Trichophyton* sp. cannot be identified via Wood’s (ultraviolet) lamp, as the fungus does not fluoresce [27]. *Trichophyton* sp. can be identified from culture by white mold on DTM culture, with the production of relatively elongate, blunt-ended macroconidia that have nearly parallel walls as well as numerous microconidia; this was the morphology observed in cases 3, 8, 9, 11, and 12 (Figure 5). The arthrospore is produced in tissue to the exclusion of conidia and is thought to be the mode of inter-animal transmission [27]. *Trichophyton* infections are reported most often in guinea pigs, and is common in dogs and cats, with other affected animal species including rabbits, hamsters, rats, chinchillas, and hedgehogs [28,30,31,32]. *Trichophyton mentagrophytes*, which commonly infects animals can be broken down into three varieties: *T. mentagrophytes* var. *mentagrophytes*, which infects rodents, dogs, horses, guinea pigs, throughout the world [27,33]; *T. mentagrophytes* var. *erinacei*, which infects European hedgehogs, dogs, and people in Europe, New Zealand, Chile, and elsewhere [27,34,35,36]; and *T. mentagrophytes* var. *quinckeanumi*, which infects mice, and rarely people in North America, Europe, and Australia [37,38]. With increased genomic data from routine diagnostic cases, outbreaks, and research being constantly compiled, there appears to be increasing complexity and granularity to the differentiation of *T. mentagrophytes* and associated species, and our understanding of this phylogenetic relatedness remains a work in progress [24,30]. 

In those cases wherein culture was not performed, next generation sequencing was attempted on genetic material extracted from formalin fixed, paraffin embedded skin samples. This provided a picture of the main fungal species prevalent within the sample as determined by read abundance and gave a picture of the species that co-occurred either as part of the infection or the microbiome of the host. The data generated indicated the presence of DNA from a diverse mixture of fungal species in the phyla *Ascomycota* (which includes *Trichophyton*) and *Basidiomycota*. Taken together, the diverse array of classes represented likely indicates that the novel method application we describe is highly sensitive. As the D1-D2 28S subunit traditionally used for fungal culture identification is a large amplicon that is not ideal for the analysis of FFPE specimens, species classification based on ITS sequences provided additional resolution to obtain species-level taxonomic assignments. It is worth noting that in two of the four FFPE samples with sufficient DNA quantity and quality, metagenomics identified *Trichophyton* sp. or *A. benhamiae*. Thus, we have shown that by applying the techniques we outlined, nucleic acids of an infected host tissue extracted from an archived FFPE sample can be used to accurately identify the etiologic agent to the species level. Deterioration of the DNA, for example, due to prolonged incubation in formalin, or tissue autolysis prior to fixation are a potential limitation. Further validation of this approach on representative sets of clinical and normal specimens for different animal species is needed. 

The genomic material from the fungi identified with the metagenomic techniques probably represent commensal organisms/members of the cutaneous microbiome of the animals as well as potentially organisms found in its immediate environment that were present during tissue fixation. Amongst the varied classes represented, *Eurotiomycetes* is of particular importance as it includes *Trichophyton* amongst its many genera of pathogens. There is a prior report of *A. benhamiae* or *T*. *mentagrophytes* in a family of NAP in a Japanese zoo, and *T*. *mentagrophytes* was isolated from a South African porcupine, though it is important to note that the South African porcupine is not a closely related species to NAP [32,39]. There is a single case report outlining cutaneous mycosis in an individual NAP due to infection with the yeast-like *Aureobasidium pullulans* [40]. A very recent paper reports a more limited infection with the geophilic (soil-associated) dermatophyte *Microsporum gypseum* in a zoo-housed porcupine [14].

In contrast to the isolated lesion from the single case of dermatophytosis in a zoo-housed NAP caused by the geophilic *M.* gypseum case, our 12 cases of wild NAP with *T. mentagrophytes* had diffuse distribution [14]. In addition, the mild inflammatory reaction, dramatic hyperkeratosis, and lack of folliculitis in this case are atypical for dermatophytosis in other mammals [41]. The common lesions of dermatophytosis in domestic animals are well-circumscribed and alopecic with variable scaling; histopathology reveals pyogranulomatous folliculitis and furunculosis with occasional kerion formation [26]. The lack of a significant inflammatory response in our cases, and the disseminated and hyperkeratotic lesions could be speculated to be related, in that poor inflammation may have allowed the spread of the infection over wide areas, with the noted hyperkeratosis the hosts’ defensive response to the fungus in lieu of an effective immune response. Susceptibility and severity of dermatophytosis is thought to depend on the pathogenicity and species of the fungus in combination with host factors including keratin composition, health status, and immune response of the host [42]. In particular, the degree of the cell-mediated immune response has alleged import: more host-adapted fungal strains may elicit relatively mild cell-mediated reactions in their primary host [42]. 

Transmission of zoophilic strains of dermatophytes occurs through direct contact between animals or with environments contaminated by infected crusts or hairs. Our 12 animals were free-ranging and admitted to wildlife rehabilitation facilities with/due to skin disease and debilitation; infections were not acquired in captivity. NAP are largely solitary animals, and the frequency of contact between animals would generally not be high; however, communal denning behavior as a matter of necessity/desperation is reported in winter months, and seen in increasing incidence with decreasing availability of suitable den sites [1,43]. In a study of nests of *T. mentagrophytes* var. *erinacei* in European hedgehogs, six infected nests were found to be inhabited by infected animals, while four uninfected nests had uninfected inhabitants [35]. Furthermore, this study found that (a) the fungus was stable in dry nest material in the laboratory for at least a year, but stable for less than a week in wet material; and (b) that four summer nests that were occupied only transiently were not infected [35]. It is our speculation that transmission occurs during winter communal denning in NAP, as is likely in European hedgehogs, and as is speculated to be the case with *Sarcoptes scabiei* transmission in NAP [1]. Even if this were true it is still remarkable that the numerous animals were infected by such genetically similar fungi, suggesting a potential species-wide immunologic susceptibility for this particular pathogen, as in scabies. It is possible that just as with white nose syndrome and chytrid fungus, dermatophytosis in NAP may be an emerging infectious disease with potential ties to climate change and anthropogenic effects. Based on the 12 cases we described here, dermatophytosis should be a differential diagnosis for hyperkeratosis and disseminated dermatopathy in wild as well as captive NAP. Our experiences with these cases also suggest that terbinafine or nitraconazole are the most effective treatment options. 

## Figures and Tables

**Table 1:** Case descriptions.

Animal #	Year	Location Found/Origin	Signalment	Treatments	Outcome (Died/Euth/Released)
1	2010	Kittery Point, ME	Juv M	Ketaconazole 20 mg/kg SID 19 days; Baytril 5 mg/kg BID 3 day; Gentak ointment BID-TID 21 days; Vetropolycin BID 3 days; Optimmune ointment SID 1 day;	Died
2	2010	Kennebunk, ME	Juv M	Ketaconazole 20 mg/kg & 30 mg/kg SID (dosage was increased); Sulfatrim 30 mg/kg BID 10 days; Gentacin ointment SID 7 days	Euthanized
3	2011	Eliot, ME	Ad M	Ketaconazole 20 mg/kg at least 4 weeks	Died
4	2012	Deerfield, NH	<1y F	*data not available*	Euthanized
5	2013	Huntington, MA	Ad F	Ketaconazole 15 mg/kg SID	Released
6	2015	Rockingham County, NH	Juv M	Ketaconazole 30 mg/kg SID; Ivermectin 0.2 mg/kg SQ	Euthanized
7	2016	Barrington, NH	M	*data not available*	Released
8	2017	North Berwick, ME	Ad M	*data not available*	Euthanized
9	2017	Wells, ME	Ad M	*data not available*	Released
10	2017	Sandbornville, NH	Ad F	ivermectin SQ; terbinafine 125 mg/mL; 30 mg/kg	Released
11	2017	Turner, ME	Ad M	*data not available*	Released
12	2017	York, ME	Ad M	nitraconazole 200 mg BID 14 d; Terbinafine 100 mg SID 32 d	Released

Abbreviations: Ad—adult; Juv—juvenile; F—female; M—male; SID—once a day; BID—twice a day; SQ—subcutaneous.

**Table 2:** Summary of diagnostic testing performed.

Animal #	Tests Performed	Culture Findings	Molecular Diagnostics (Specimen Tested—Result)	Additional Necropsy Findings
1	Necropsy; FFPE metagenomics	Culture not performed	FFPE—primarily *Arthroderma benhamiae*	(1) Fibrinonecrotizing neutrophilic pneumonia; (2) Chronic cerebrocortical necrosis (suspected infarct); (3) Hepatic and esophageal subacute fibrinoid phlebitis; (4) Marked intestinal cestodiasis; (5) Marked colonic nematodiasis
2	Necropsy; FFPE metagenomics	Culture not performed	FFPE—poor DNA yield/ no amplification	(1) Subacute lymphohistiocytic interstitial myocarditis; (2) Eosinophilic and neutrophilic bronchopneumonia; (3) Marked intestinal cestodiasis
3	Necropsy; Fungal culture; D1-D2 large subunit RNA PCR and sequencing	*Trichophyton* sp.	culture—*Arthroderma benhamiae*	(1) Incisor overgrowth & marked molar wear; (2) Skeletal myofiber atrophy; (3) Rib fractures; (4) Marked intestinal cestodiasis; (5) Moderate colonic nematodiasis; (6) Subacute lymphohistiocytic myocarditis; (7) Thalamic gliosis and sclerosis with minimal encephalitis
4	Biopsy; FFPE metagenomics	Culture not performed	FFPE—poor DNA yield/ no amplification	n/a—biopsy only
5	Biopsy; FFPE metagenomics	Culture not performed	FFPE—primarily *Resinicium furfuraceum*	n/a—biopsy only
6	Necropsy; dermatophyte test media culture (DTM); FFPE metagenomics	DTM +	FFPE—primarily *Puccinia coronate*	(1) Granulomatous peritonitis with abdominal perforation, intestinal entrapment and rupture (presumptive trauma); (2) Moderate intestinal cestodiasis; (3) Moderate colonic nematodiasis; (4) Chronic cerebrocortical astrogliosis and microgliosis; (5) Subacute hepatocellular necrosis (presumptive larval migration)
7	Biopsy; FFPE metagenomics	Culture not performed	FFPE—primarily *Trichophyton* sp. (28S), *Arthroderma benhamiae* (ITS)	n/a—biopsy only
8	Necropsy; culture; D1-D2 large subunit RNA PCR and sequencing	*Trichophyton* sp.	culture—*Arthroderma benhamiae*	(1) Chronic granulomatous and eosinophilic capsular splenitis; (2) Marked intestinal cestodiasis; (3) Marked colonic nematodiasis
9	Biopsy; culture; D1-D2 large subunit RNA; metagenomics on culture	*Trichophyton* sp.	culture—*Arthroderma benhamiae*	n/a—biopsy & culture only
10	Biopsy; culture; D1-D2 large subunit RNA; metagenomics on culture	Mixed	culture—mixedprimarily *Scopulariopsis candida*	n/a—biopsy & culture only
11	Biopsy; culture; D1-D2 large subunit RNA; metagenomics on culture	*Trichophyton* sp.	culture—*Arthroderma benhamiae*	n/a—biopsy & culture only
12	Biopsy; culture; D1-D2 large subunit RNA; metagenomics on culture	*Trichophyton* sp.	culture—*Arthroderma benhamiae*	n/a—biopsy & culture only

Abbreviations: FFPE—formalin-fixed, paraffin-embedded tissue; n/a—not applicable.
